# Impact of Diabetes Mellitus on the Prognosis of Patients with Hepatocellular Carcinoma after Curative Hepatectomy

**DOI:** 10.1371/journal.pone.0113858

**Published:** 2014-12-01

**Authors:** Yan-Yan Wang, Shan Huang, Jian-Hong Zhong, Yang Ke, Zhe Guo, Jia-Qi Liu, Liang Ma, Hang Li, Bing-Ning Ou, Le-Qun Li

**Affiliations:** 1 Department of Hepatobiliary Surgery, Affiliated Tumor Hospital of Guangxi Medical University, Nanning, China; 2 Department of Ultrasound, Affiliated Tumor Hospital of Guangxi Medical University, Nanning, China; 3 Pharmaceutical College, Guangxi Medical University, Nanning, China; Yonsei University College of Medicine, Korea, Republic Of

## Abstract

***Background:*** The influence of diabetes mellitus (DM) on the prognosis of patients with hepatocellular carcinoma (HCC) remains controversial. Here we investigated the impact of DM on the prognosis of such patients after curative hepatectomy.

***Methods:*** A consecutive cohort of 505 patients with HCC (134 with DM, 371 without) underwent curative hepatectomy were retrospectively evaluated. Postoperative morbidity and mortality, overall survival (OS) and disease-free survival (DFS) were compared between patients with or without DM. Independent prognostic predictors were identified using the Cox proportional hazards model.

***Results:*** Patients with or without DM showed similar morbidity and 30- and 90- day mortality after curative hepatectomy (all *P*>0.05), as well as similar DFS at 1, 3, 5 years (*P* = 0.781). However, the group of patients with DM showed significantly lower OS at 1, 3, 5 years than the group without DM (*P* = 0.038). Similar results were obtained in the propensity-matched cohort. Cox multivariate analysis identified DM as an independent predictor of poor OS, but not of poor DFS. We repeat compared OS and DFS for DM and non-DM subgroups defined according to the presence or absence of hepatitis B virus infection and cirrhosis. Similar results were obtained in all subgroups except the non-cirrhotic subgroup which showed patients with and without DM had similar OS.

***Conclusions:*** DM does not significantly affect the postoperative morbidity or mortality or the DFS of patients with HCC after curative hepatectomy. It is, however, associated with significantly lower OS, especially in patients with cirrhosis.

## Introduction

Hepatocellular carcinoma (HCC) is one of the most common malignancies worldwide, and its incidence is increasing in many countries [1]. Hepatectomy is a radical therapy for HCC that can be highly effective for immediate improvement. However, the prognosis of many patients remains poor because of the high recurrence rate [2]–[4].

Cirrhosis occurs in 80 to 90% of patients with HCC [5], and it increases the risk of the disease [6]. Cirrhosis has been strongly associated with impaired glucose tolerance or diabetes mellitus (DM) due to defects in glucose metabolism in the damaged liver [7]–[9]. As a result, a substantial proportion of patients with HCC also have DM [10], [11]. In fact, recent epidemiological studies suggest that DM increases the risk of HCC [12]–[14].

Whether DM also adversely affects the prognosis of patients with HCC remains controversial. Some retrospective studies identified DM as an independent predictor of poor prognosis in patients with HCC after hepatectomy [15]–[17]. On the other hand, Poon and cowokers [11] came to the opposite opinion, reporting that DM does not increase HCC recurrence or affect long-term survival. The discrepancies among these studies may be due, at least in part, to their relatively small cohorts and to nonrandom differences in baseline clinical factors between patient groups. It is important to resolve whether DM affects the prognosis of HCC patients in order to guide long-term disease management.

Here we performed a retrospective analysis of a relatively large cohort of patients at a regional HCC treatment center in southeast China. Our goal was to assess whether DM affects post-hepatectomy prognosis of HCC patients. In order to control for numerous possible confounders of HCC prognosis, we also analyzed outcomes after pairing patients with and without DM using propensity score analysis.

## Patients and Methods

### Ethics Statements

This retrospectively study was approved by the Ethics Committee of the Affiliated Tumor Hospital of Guangxi Medical University, and it was performed according to the Declaration of Helsinki 2013 edition. Written informed consent was obtained from patients, and patient records or information was anonymized prior to analysis.

### Patients

All patients who underwent curative hepatectomy for primary HCC at the Affiliated Tumor Hospital of Guangxi Medical University between June 2003 and February 2011 were eligible for inclusion in this study. Patients were excluded if they (a) were initially treated for HCC at other centers, (b) underwent transarterial chemoembolization or other antitumor therapies before surgery, or (c) suffered from additional malignancies simultaneously. Patients data were originally collected prospectively in a computer database and then analyzed retrospectively for this study.

### Diagnosis and Definitions

DM was diagnosed as a fasting plasma glucose level of ≥7.0 mmol/L (126 mg/dL), or a plasma glucose level of ≥11.1 mmol/L (200 mg/dL) at 2 h in a 75-g oral glucose tolerance test, or typical DM symptoms together with a casual plasma glucose level of ≥11.1 mmol/L (200 mg/dL) [18]. A fasting glucose concentration between 5.6 and 11.1 mmol/L was maintained preoperatively in our cohort through a combination of diet and oral antidiabetic drugs or subcutaneous injection of insulin. The plasma glucose level was monitored carefully during and after the operation to ensure that it remained below 11.1 mmol/L.

Diagnoses of HCC and liver cirrhosis were confirmed after hepatectomy by histopathological examination of resected liver tissue. HCC stage was determined according to the Barcelona Clinic Liver Cancer (BCLC) staging system [19]. Curative hepatectomy was defined as complete resection of the visible tumor and no tumor residual revealed by imaging tests within 1 month after resection. Major resection was defined as the resection of three or more segments according to Couinaud's classification [20]. Liver failure was defined as persistently elevated serum total bilirubin (>100 mmol/L) or prolonged prothrombin time (>24 s), or hepatic encephalopathy [21].

### Treatment and Follow-up

Our cohort was treated by hepatectomy based on the following indications: (a) good performance status, with an Eastern Cooperative Oncology Group score of 0–2; (b) good cardiopulmonary function, without severe disease in other important organs or systems; (c) Child-Pugh grade A or B liver function; (d) no extrahepatic metastasis; and (e) adequate residual liver volume (30% for patients without cirrhosis and 50% for patients with cirrhosis or other severe liver diseases) based on volumetric computed tomography [22], [23]. Hepatectomy was performed as described [24].

After hepatectomy, all patients in our cohort were followed up at 1, 3, 6, 9, and 12 months later, and then every 6 months thereafter. The following tests were performed at each follow-up visit: serum alpha-fetal protein (AFP), serum markers of hepatitis B virus (HBV) infection, liver function, prothrombin time, abdominal ultrasonography, chest radiography, and enhanced computed tomography or magnetic resonance imaging. Tumor recurrence, which was defined to include intra- and extrahepatic recurrence, was diagnosed based on the combination of elevated AFP level and typical findings by enhanced computed tomography or magnetic resonance imaging.

### Propensity Score Analysis

The propensity score analysis was used to reduce the bias in patient selection in observational studies [25]–[27]. It seeks to eliminate confounding similarly to randomization, by creating comparison arms with similar distributions of measured baseline covariates [27]. The following variables were entered into the propensity model: gender, age, body mass index, hepatitis B surface antigen, hepatitis C antibody, AFP, total bilirubin, albumin, alanine aminotransferase (ALT), γ-glutamyl transferase (GGT), creatinine, Blood urea nitrogen, creatinine clearance rate, total cholesterol, sodium, prothrombin time, platelet count, ascites, comorbidities, tumor capsule status, macrovascular invasion, tumor size, tumor number, tumor cell differentiation, type of resection, surgery duration, blood loss and blood transfusions. Data for these variables were fit by logistic regression to generate a continuous propensity score ranging from 0 to 1. One-to-one nearest-neighbor matching between patients with and without DM was performed using a 0.1 caliper width, generating score-matched pairs for subsequent analysis [28], [29].

### Statistics Analysis

Normally distributed data were expressed as mean ± SD, while non-normally distributed data were expressed as median (range). The significance of intergroup differences in continuous data was assessed using the *t* test or Mann-Whitney *U* test, while that of differences in categorical data was assessed using the chi-squared test or Fisher's exact test (2-tailed). The Kaplan-Meier method was used to estimate overall survival (OS) and disease-free survival (DFS) and the log-rank test was used to compare differences. Multivariate analysis was performed using the Cox proportional hazards model to identify independent prognostic factors. All statistical analyses were conducted with SPSS 19.0 (Chicago, IL, USA), using 2-tailed *P*<0.05 as the threshold for statistical significance.

## Results

### Study Population

Between June 2003 and February 2011, 937 patients underwent hepatectomy for HCC in the Affiliated Tumor Hospital of Guangxi Medical University. Of these, 785 (83.8%) underwent curative hepatectomy, and the remaining 152 (16.2%) not. Among the 785 patients with curative hepatectomy, 280 (35.7%) were excluded because they (a) were initially treated for HCC at other centers (238 patients, 30.3%), (b) underwent transarterial chemoembolization or other antitumor therapies before surgery (22 patients, 2.8%), or (c) suffered from additional malignancies simultaneously (20 patients, 2.6%). In the end, 505 (64.3%) patients were enrolled in this study.

### Clinicopathological Data

Among the 505 patients, 134 (26.5%) were diagnosed with DM and were included in the DM group, while the remaining 371 (73.5%) were included in the non-DM group. Among the 134 DM patients, 46 controlled their glucose level through subcutaneous injection of insulin, and the remaining 88 were with oral antidiabetic drugs (metformin, acarbose, sulfonylurea, etc). Pre- and intraoperative characteristics of both groups are shown in Table 1. The parameters of the two groups were similar across numerous variables, including gender composition, prevalences of HBV and hepatitis C virus (HCV) infection, blood urea nitrogen, creatinine clearance rate, serum sodium, tumor capsule status, presence of macrovascular invasion, tumor number, tumor cell differentiation, tumor stages, operation time, intraoperative blood loss, etc. However, some parameters of the two groups were unbalanced: age, body mass index, AFP, total bilirubin, albumin, ALT, GGT, creatinine, total cholesterol, prothrombin time, platelet count, proportions with Child-Pugh A liver function and major resection, presence of ascites, incidences of liver cirrhosis and hypertention, and proportion of patients who required blood transfusions.

**Table 1:** Clinicopathologic characteristics of patients with or without diabetes mellitus treated for hepatocellular carcinoma by curative hepatectomy.

	Before propensity matching (*n* = 505)			After propensity matching (*n* = 198)		
Characteristic	DM (*n* = 134)	Non-DM (*n* = 371)	*P*	DM (*n* = 99)	Non-DM (*n* = 99)	*P*
Male, *n* (%)	122 (91)	325 (88)	0.284	90 (91)	89 (90)	0.809
Age, yr	56.0±8.8	48.3±11.6	<0.001	54.3±8.8	52.5±10.5	0.260
Body mass index, kg/m^2^	23.4±3.6	22.2±3.3	<0.001	22.8±3.9	23.2±3.2	0.410
Positive HBsAg, *n* (%)	108 (81)	322 (87)	0.084	84 (85)	84 (85)	1.000
Positive anti-HCV, *n* (%)	2 (1)	6 (2)	1.000	0 (0)	1 (1)	1.000
AFP, *n* (%), ng/mL						
≥400	30 (22)	127 (34)	0.011	24 (24)	26 (26)	0.744
<400	104 (78)	244 (66)		75 (76)	73 (74)	
Total bilirubin, µmol/L	16.9±11.5	14.2±6.6	0.010	14.5±6.1	14.7±6.7	0.911
Albumin, g/L	38.9±5.8	40.5±4.8	0.002	40.0±6.0	40.1±5.7	0.925
ALT, U/L	45 (12–294)	39 (3–504)	0.002	44 (17–294)	41 (3–399)	0.463
GGT, U/L	94 (13–1429)	57 (10–433)	<0.001	61 (17–388)	58 (10–433)	0.459
Creatinie, µmol/L	77 (45–316)	81 (37–201)	0.018	78 (52–149)	80 (37–117)	0.797
Blood urea nitrogen, mmol/L	5.0 (2.1–17.2)	5.0 (2.2–11.6)	0.503	5.3 (2.1–9.8)	5.0 (2.7–9.5)	0.636
Ccr, ml/min	91 (39–150)	92 (47–146)	0.726	91 (59–150)	89 (55–138)	0.364
Total cholesterol, mmol/L	4.7±1.0	4.3±0.7	0.018	4.5±1.2	4.4±1.0	0.559
Sodium, mmol/L	140.3±2.9	140.8±2.4	0.181	140.5±2.8	140.9±2.4	0.347
Prothrombin time, sec	13.4 (10.0–22.4)	12.8 (10.0–24.0)	<0.001	13.1 (10.4–22.4)	13.0 (10.2–21.0)	0.369
Platelet count, 10^9^/L	127 (31–352)	176 (31–610)	<0.001	144 (31–352)	151 (32–367)	0.734
Child-Pugh A, *n* (%)	119 (88.8)	358 (96.5)	0.001	92 (93)	94 (95)	0.551
Ascites, *n* (%)	34 (25.4)	62 (16.7)	0.029	23 (23)	23 (23)	1.000
Comorbidities, *n* (%)						
Cirrhosis	104 (78)	234 (63)	0.002	74 (75)	71 (72)	0.630
Hypertention	23 (17.2)	29 (7.8)	0.002	15 (15)	13 (13)	0.683
Heart disease	2 (1.5)	2 (0.5)	0.618	1 (1)	0 (0)	1.000
Cerebrovascular disease	3 (2.2)	5 (1.3)	0.761	3 (3)	2 (2)	1.000
Renal disease	6 (4.5)	11 (3.0)	0.580	4 (4)	3 (3)	1.000
Tumor capsule, *n* (%)						
Complete	82 (61)	214 (58)	0.479	61 (62)	60 (61)	0.884
Incomplete	52 (39)	157 (42)		38 (38)	39 (39)	
Macrovascular invasion, *n* (%)	20 (15)	59 (16)	0.789	15 (15)	13 (13)	0.683
Tumor size, cm	4.0 (1.5–16.0)	5.5 (1.0–18.0)	0.004	5.0 (2.0–16.0)	5.0 (1.0–14.0)	0.734
Tumor number, *n* (%)						
<3	117 (87)	323 (87)	0.941	88 (89)	88 (89)	1.000
≥3	17 (13)	48 (13)		11 (11)	11 (11)	
Differentiation degree, *n* (%)						
Well	15 (11)	44 (12)	0.073	12 (12)	12 (12)	0.545
Moderately	67 (50)	220 (59)		52 (53)	57 (58)	
Poorly	52 (39)	107 (29)		35 (35)	30 (30)	
BCLC stage, *n* (%)						
0 and A	66 (49)	169 (46)	0.462	48 (48)	50 (51)	0.776
B and C	68 (51)	202 (54)		51 (52)	49 (49)	
Major resection, *n* (%)	11 (8.2)	68 (18.3)	0.006	9 (9)	13 (13)	0.366
Operation time, min	155 (70–385)	150 (60–495)	0.408	165 (100–385)	165 (80–495)	0.644
Blood loss, mL	300 (50–3000)	250 (20–8400)	0.239	300 (50–3000)	300 (20–2500)	0.960
Required blood transfusion, *n* (%)	30 (22)	44 (12)	0.003	19 (19)	17 (17)	0.712
30-d mortality, *n* (%)	0 (0)	1 (0.3)	1.000	0 (0)	0 (0)	1.000
90-d mortality, *n* (%)	1 (0.7)	7 (1.9)	0.615	1 (1.0)	3 (3.0)	0.613
Postoperative complications, *n* (%)	49 (36.6)	110 (29.6)	0.139	35 (35.4)	31 (31.1)	0.546

Data are mean ± SD or median (range).

Abbreviations: ALT, alanine aminotransferase; AFP, alpha-fetoprotein; BCLC, Barcelona Clinic Liver Cancer; Ccr, creatinine clearance rate; DM, diabetes mellitus; GGT, γ-glutamyl transferase; HBsAg, hepatitis B surface antigen; HCV, hepatitis C virus.

Propensity score analysis based on variables associated with prognosis indentified 99 matched pairs of patients from each group. In the propensity-matched cohort, there were no significant differences in pre- or intraoperative characteristics between DM and non-DM patients (Table 1).

### Mortality and Morbidity

In the entire study cohort, the DM and non-DM groups showed similar frequency of postoperative complications and similar 30- and 90- day mortality (Table 1). Comparison of the distribution of specific postoperative complications between the two groups of patients (Table S1) showed that the only difference was the proportion of patients with ascites, which was significantly higher in the DM group (13.4%) than in the non-DM group (6.7%, *P* = 0.017). In both groups, pleural effusion was the most frequent complication (17.2% vs 13.5%, *P* = 0.298). In the propensity-matched cohort, we found similar results.

In addition, we compared the severity of postoperative complications between DM and non-DM patients in the propensity-matched cohort using the Clavien-Dindo classification [30] of surgical complications. No significant difference was found in the severity of postoperative complications between the two groups of patients (*P* = 0.218; Table 2).

**Table 2:** Severity of postoperative complications assessed by the Clavien-Dindo classification in a propensity-matched cohort of patients with or without diabetes mellitus (DM) treated for hepatocellular carcinoma by curative hepatectomy.

	No. (%) patients		
Severity	DM (*n* = 99)	Non-DM (*n* = 99)	*P*
Grade I	18 (18.2)	23 (23.2)	0.218
Grade II	22 (22.2)	20 (20.2)	
Grade III-a	10 (10.1)	8 (8.1)	
Grade III-b	1 (1.0)	0 (0)	
Grade IV-a	1 (1.0)	1 (1.0)	
Grade IV-b	1 (1.0)	0 (0)	
Grade V	0 (0)	0 (0)	

### Overall Survival

**Figure 1:**
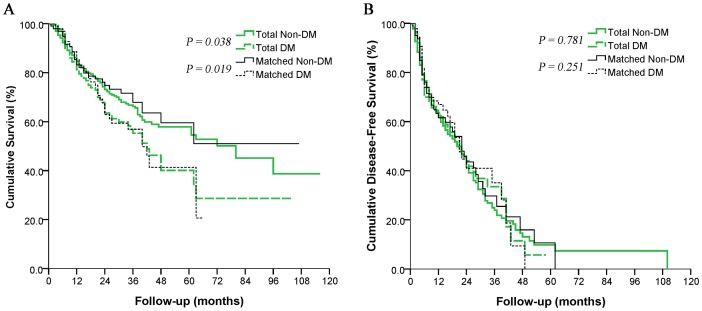
Overall survival (*A*) and disease-free survival (*B*) in diabetic and non-diabetic patients with hepatocellular carcinoma after curative hepatectomy. Separate curves are shown for the entire cohort and for the propensity-matched cohort.

The entire cohort was followed up for a median period of 31 months (range, 1-116). During follow-up, 56 patients (41.8%) died in the DM group, compared to 120 (32.3%) in the non-DM group. OS was significantly lower in the DM group than in the non-DM group (*P* = 0.038; Fig. 1A). OS at 1, 3, or 5 years was 81.3%, 55.3%, and 34.4% in the DM group, compared to 84.7%, 66.6%, and 54.2% in the non-DM group.

Univariate analysis identified the following significant prognostic factors for poor OS: DM, serum AFP level ≥400 ng/ml, serum albumin level <35 g/L, serum GGT level ≥50 U/L, ascites, incomplete tumor capsule, macrovascular invasion, tumor size ≥10 cm, tumor number ≥3, poor degree of tumor cell differentiation, and operation time >180 min (Table S2). Multivariate analysis identified the following factors as independent predictors for poor OS: DM, serum AFP level ≥400 ng/ml, serum albumin level <35 g/L, serum GGT level ≥50 U/L, incomplete tumor capsule, macrovascular invasion, tumor size ≥10 cm, tumor number ≥3, and poor degree of tumor cell differentiation (Table 3).

**Table 3:** Multivariate analysis to identify factors predicting poor overall survival and disease-free survival of patients with hepatocellular carcinoma after curative hepatectomy.*

	Hazard Ratio	95% *CI*	*P*
*Overall Survival*			
Diabetes mellitus	1.482	1.044–2.104	0.028
AFP (≥400 ng/mL)	1.603	1.108–2.319	0.012
Albumin (<35 g/L)	1.634	1.035–2.577	0.035
GGT (≥50 U/L)	1.891	1.245–2.872	0.003
Tumor capsule (Incomplete)	1.553	1.073–2.247	0.020
Macrovascular invasion	2.333	1.561–3.486	<0.001
Tumor size (≥10 cm)	1.112	1.012–1.223	0.027
Tumor number (≥3)	2.431	1.596–3.702	<0.001
Differentiation degree (Poorly)	2.380	1.266–4.475	<0.001
*Disease-free survival*			
Diabetes mellitus	0.878	0.624–1.237	0.458
AFP ( ≥400 ng/mL)	1.399	1.011–1.937	0.043
Albumin (<35 g/L)	1.425	0.944–2.151	0.092
GGT (≥50 U/L)	1.450	1.040–2.020	0.028
Tumor capsule (Incomplete)	1.563	1.136–2.146	0.006
Macrovascular invasion	1.638	1.124–2.388	0.010
Tumor size (≥10 cm)	1.098	1.000–1.205	0.049
Tumor number (≥3)	2.138	1.453–3.147	<0.001
Differentiation degree (Poorly)	1.814	1.102–2.984	0.019

*Calculated using data from all patients in the original cohort (without propensity score matching).

Abbreviations: AFP, alpha-fetoprotein; CI, confidence interval, GGT, γ-glutamyl transferase.

Analysis of the propensity-matched cohort showed that, as for the entire cohort, OS at 1, 3, or 5 years was significantly lower in the DM group (82.9%, 56.9%, and 41.4%) than in the non-DM group (83.2%, 67.9%, and 59.6%; *P* = 0.019; Fig. 1A).

### Disease-free Survival

In the entire cohort, 232 patients (45.9%) experienced tumor recurrence during follow-up, including 57 (42.5%) in the DM group and 175 (47.2%) in the non-DM group. Of these 232 patients, 203 (87.5%) presented initially with intrahepatic recurrence, while 29 (12.5%) presented with either extrahepatic recurrence or concurrent intra- and extrahepatic recurrence. DFS at 1, 3, or 5 years was similar between the DM group (63.4%, 28.7%, and 5.7%) and non-DM group (62.4%, 23.9%, and 7.4%; *P* = 0.781; Fig. 1B). Patients with recurrence were treated with transarterial chemoembolization (169 patients, 72.8%), reoperation (22, 9.5%), radiofrequency ablation (15, 6.5%) or other treatments (26, 11.2%).

Univariate analysis identified the following significant prognostic factors for poor DFS: serum AFP level ≥400 ng/ml, serum GGT level ≥50 U/L, incomplete tumor capsule, macrovascular invasion, tumor size ≥10 cm, tumor number ≥3, poor degree of tumor cell differentiation , and operation time >180 min (Table S3). Multivariate analysis identified the following factors as independent predictors for poor DFS: serum AFP level ≥400 ng/ml, serum GGT level ≥50 U/L, incomplete tumor capsule, macrovascular invasion, tumor size ≥10 cm, tumor number ≥3, and poor degree of tumor cell differentiation (Table 3).

Analysis of the propensity-matched cohort showed that, as for the entire cohort, DFS at 1, 3, or 5 years was similar between the DM group (67.0%, 35.1%, and 0%) and non-DM group (61.6%, 29.7%, and 10.6%; *P* = 0.251; Fig. 1B).

### Subgroup Analysis of the Entire Study Patients

To explore the underlying cause why DM may affect the OS, we compared OS and DFS for DM and non-DM subgroups defined according to the presence or absence of HBV infection and cirrhosis.

**Figure 2:**
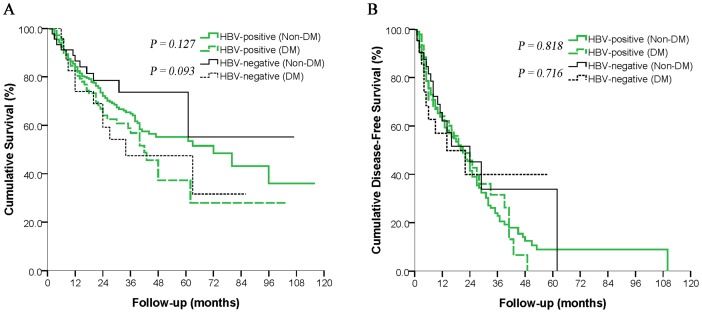
Subgroup analyses of the overall survival (*A*) and disease-free survival (*B*) of diabetic and non-diabetic patients with hepatocellular carcinoma after curative hepatectomy according to the presence or absence of hepatitis B virus (HBV) infection. Patients with hepatitis C virus infection were excluded.

**Figure 3:**
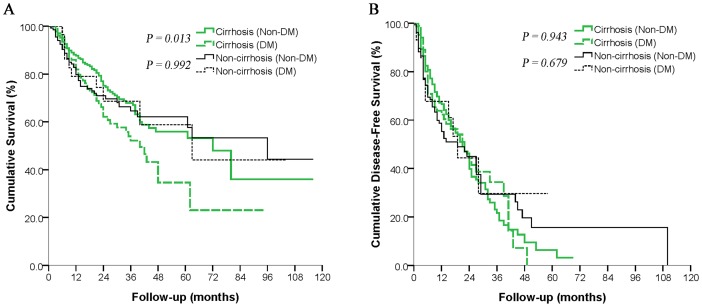
Subgroup analyses of the overall survival (*A*) and disease-free survival (*B*) of diabetic and non-diabetic patients with hepatocellular carcinoma after curative hepatectomy according to the presence or absence of cirrhosis.

After excluding 8 patients infected with HCV, we divided HBV-positive patients into DM and non-DM groups and did the same with HBV-negative patients. Among HBV-positive patients, patients with DM had lower OS than patients without DM, although this difference did not achieve statistical significance (*P* = 0.127; Fig. 2A). A similar result was obtained among the subgroups of HBV-negative patients (*P* = 0.093; Fig. 2A). DFS did not differ significantly between DM or non-DM patients in either HBV subgroup (Fig. 3B).

In a separate analysis, we divided cirrhotic patients into DM and non-DM subgroups and did the same with non-cirrhotic patients. OS was significantly lower in cirrhotic patients with DM than in cirrhotic patients without DM (*P* = 0.013; Fig. 3A). OS did not, however, differ between DM and non-DM patients without cirrhosis (*P* = 0.992; Fig. 3A). DFS did not differ significantly between DM or non-DM patients in either cirrhosis subgroup (Fig. 3B).

## Discussion

DM is a common comorbidity in HCC patients, and growing studies indicated that it is associated with increased risk of HCC and other malignancies [13], [31]–[33]. Some authors suggest that DM can also significantly worsen prognosis of HCC patients after hepatectomy, while others disagree. The frequency of DM among HCC patients argues for careful assessment of whether and how it affects clinical outcomes. Here we examined short- and long-term outcomes in a relatively large cohort of HCC patients (26.5% with DM) treated by curative hepatectomy at a large regional HCC treatment center in southeast China. Analysis of the entire cohort, as well as of a propensity-matched cohort without baseline variations that might mask the effects of DM, suggests that DM does not increase postoperative morbidity or mortality or DFS, but that it does reduce long-term OS, especially in patients with cirrhosis.

Risk of postoperative morbidity and mortality is an important factor when clinicians decide whether or not hepatectomy is suitable for a given patient [34]. Studies of a total of 551 patients from Japan suggested that DM increases postoperative morbidity, but not postoperative mortality, after hepatectomy [16], [35]. In the present study, however, we found similar morbidity and 30- and 90-day mortality between patients with and without DM. Moreover, we found the severity of postoperative complications to be similar between the two groups of patients based on the Clavien-Dindo classification. This discrepancy may be due to the fact that the Japanese studies were conducted at least 16 years ago, and improvements in surgical technique and perioperative care may have helped reduce the adverse effects of DM on postoperative outcome. In fact, our results are consistent with those of more recent studies in Hong Kong [11] and the US [36], whichwhich reported that DM does not increase the risk of postoperative complications. We conclude that DM should not be considered a negative factor when selecting HCC patients for hepatectomy.

At the same time, our evidence suggests that care should be taken to prevent ascites in HCC patients with DM. This complication was significantly more prevalent in patients with DM than in patients without it (13.4% vs 6.7%, *P* = 0.017). The reason for this difference is unclear, but it may be explained by the high prevalence of nephropathy in DM patients [37]. In one Egyptian study of 1661 diabetic outpatients, 25.4% were found to have microalbuminuria, considered the earliest clinical sign of diabetic nephropathy [38]. Excessive albumin loss through the urine may accelerate the progression of hypoproteinemia, leading to ascites. Our results suggest that, while DM does not affect the safety of hepatectomy in HCC patients, it should signal to clinicians to take special precautions to prevent postoperative ascites.

Consistent with previous studies [15]–[17], [23], [39], [40], we found that DM significantly lowered OS of patients after curative hepatectomy in both our entire cohort and our propensity-matched cohort. However, Poon *et al.* [11] reported that DM does not significantly influence the OS of HCC patients. Notably, several clinicopathological factors differed significantly between their DM and non-DM groups at baseline. These differences may have affected prognosis, masking the influence of DM.

How DM may reduce the OS of HCC patients remains unclear. One straightforward mechanism would be that DM increases risk of recurrence, since recurrence remains the most significant problem for HCC patients after curative surgery [2]–[4], [41]. Indeed, Ikeda *et al.* [16] reported that HCC patients with DM had significantly lower DFS after hepatectomy than patients without DM. However, the results of the present study does not support this explanation: DFS even at 5 years was similar between patients with and without DM. Still, it is possible that our discrepancy from Ikeda *et al.* [16] is not a real difference but rather reflects the prognostic influence of hepatitis virus infection. While 85% of our cohort were HBV-positive, 74% of the cohort of Ikeda *et al.* [16] were HCV-positive, and evidence suggests that DM is a risk factor for recurrence of HCV-related HCC but not of HBV-related HCC [15]. In fact, a study of 525 HCC patients in Hong Kong in which 83% had HBV but only 3% had HCV concluded, like us, that DM does not affect recurrence risk after hepatectomy [11].

To separate the prognostic effects of DM from those of HBV infection, considered a much stronger oncogenic stimulus than DM [42], we compared DFS between patients with and without DM for two separate subgroups: those positive for HBV and those negative for the virus. DFS did not differ significantly with DM status in either subgroup (Fig. 2B). We conclude that our finding of lower OS for HCC patients with DM is not due to an effect of DM on recurrence.

Subgroup analysis based on the presence or absence of cirrhosis showed that DM reduced OS in cirrhotic HCC patients but not in non-cirrhotic ones (Fig. 3A), although DFS was similar for patients with or without DM in both subgroups (Fig. 3B). The differential effects of DM on cirrhotic and non-cirrhotic HCC patients may indicate that DM reduces OS by exacerbating existing liver damage. This explanation would be consistent with several studies establishing a link between DM and liver fibrosis [43] and accelerated fibrosis progression [44]. The high glucose levels and hyperinsulinemia observed in most DM patients can accelerate the progression of liver fibrosis by up-regulating the expression of connective tissue growth factor [45]. This fibrosis and liver injury can be exacerbated by the increased production of reactive oxygen species associated with hyperinsulinemia and insulin resistance [46], potentially leading to liver failure. Thus, DM may reduce the OS of HCC patients by exacerbating existing liver fibrosis, resulting in severe liver failure. DM did not reduce OS among our non-cirrhotic HCC patients, perhaps because these patients take longer to progress to liver failure. Therefore, for diabetic HCC patients, good control of blood glucose levels and aggressive treatment to preserve liver function may prolong their survival.

Despite its insights, the present study has some limitations. First, 85% of our cohort had chronic HBV infection, unlike typical HCC patient populations in most Western countries or Japan. Therefore, our findings may not extrapolate to all HCC patient populations. Second, although our propensity score analysis balanced pre- and intraoperative variables between our DM and non-DM groups, some subtle biases can not be completed eliminated. Third, we were unable to compare the causes of death between DM and non-DM patients because it is often difficult to clearly distinguish whether the cause is tumor recurrence or liver failure. These limitations argue for further studies to explore in greater detail the prognostic role of DM in HCC.

In conclusion, the present study suggests that DM does not significantly affect the postoperative morbidity or mortality or the DFS of patients with HCC after curative hepatectomy. It is, however, associated with significantly lower OS, especially in patients with cirrhosis.

## Supporting Information Legends

Table S1 **Types and frequencies of complications of patients with or without diabetes mellitus (DM) treated for hepatocellular carcinoma by curative hepatectomy.**
(DOC)

Table S2 **Univariate analysis to identify factors affecting overall survival of patients with hepatocellular carcinoma after curative hepatectomy.**
(DOC)

Table S3 **Univariate analysis to identify factors affecting disease-free survival of patients with hepatocellular carcinoma after curative hepatectomy.**
(DOC)
