# Supplementary material for: Impact of Diabetes Mellitus on the Prognosis of Patients with Hepatocellular Carcinoma after Curative Hepatectomy
Source: PLoS One. 2014 Dec 1;9(12):e113858. doi: 10.1371/journal.pone.0113858 (PMC4250061; doi:10.1371/journal.pone.0113858)
Supplement: Table S1 — Types and frequencies of complications of patients with or without diabetes mellitus (DM) treated for hepatocellular carcinoma by curative hepatectomy. (DOC) [file pone.0113858.s001.doc]

**Table S1.** Types and frequencies of complications of patients with or without diabetes mellitus (DM) treated for hepatocellular carcinoma by curative hepatectomy.

| Complication | Before propensity matching (*n* = 505) | | |  | After propensity matching (*n* = 198) | | |
| --- | --- | --- | --- | --- | --- | --- | --- |
|  | DM (*n* = 134) | Non-DM (*n* = 371) | *P* |  | DM (*n* = 99) | Non-DM (*n* = 99) | *P* |
| Pleural effusion, *n* (%) | 23 (17.2) | 50 (13.5) | 0.298 |  | 17 (17.2) | 14 (14.1) | 0.557 |
| Ascites, *n* (%) | 18 (13.4) | 25 (6.7) | 0.017 |  | 15 (15.2) | 6 (6.1) | 0.038 |
| Pulmonary infection, *n* (%) | 6 (4.5) | 21 (5.7) | 0.602 |  | 4 (4.0) | 6 (6.1) | 0.516 |
| Bile leakage, *n* (%) | 4 (3.0) | 6 (1.6) | 0.540 |  | 3 (3.0) | 1 (1.0) | 0.613 |
| Postoperative abdominal bleeding, *n* (%) | 4 (3.0) | 9 (2.4) | 0.964 |  | 2 (2.0) | 3 (3.0) | 1.000 |
| Wound infection, *n* (%) | 3 (2.2) | 6 (1.6) | 0.932 |  | 1 (1.0) | 2 (2.0) | 1.000 |
| Liver failure, *n* (%) | 4 (3.0) | 5 (1.3) | 0.219 |  | 2 (2.0) | 2 (2.0) | 1.000 |
| Abdominal infection, *n* (%) | 2 (1.5) | 7 (1.9) | 1.000 |  | 1 (1.0) | 3 (3.0) | 0.613 |
| Intestinal obstruction, *n* (%) | 0 (0) | 3 (0.6) | 0.569 |  | 0 (0) | 1 (1.0) | 1.000 |
| Cholangitis, *n* (%) | 1 (0.7) | 2 (0.5) | 1.000 |  | 1 (1.0) | 1 (1.0) | 1.000 |
| Total events/patients | 65/49 | 134/110 | - |  | 46 /35 | 39/31 | - |
