# Supplementary material for: Impact of Diabetes Mellitus on the Prognosis of Patients with Hepatocellular Carcinoma after Curative Hepatectomy
Source: PLoS One. 2014 Dec 1;9(12):e113858. doi: 10.1371/journal.pone.0113858 (PMC4250061; doi:10.1371/journal.pone.0113858)
Supplement: Table S2 — Univariate analysis to identify factors affecting overall survival of patients with hepatocellular carcinoma after curative hepatectomy. (DOC) [file pone.0113858.s002.doc]

**Table S2.** Univariate analysis to identify factors affecting overall survival of patients with hepatocellular carcinoma after curative hepatectomy.*

| Variable | *n* | 3-yr OS (%) | 5-yr OS (%) | *P* |
| --- | --- | --- | --- | --- |
| Gender |  | | | |
| Male | 447 | 63.9 | 51.3 | 0.132 |
| Female | 58 | 54.9 | 45.3 |  |
| Age, yr |  | | | |
| <60 | 400 | 63.8 | 53.7 | 0.188 |
| ≥60 | 105 | 59.1 | 41.1 |  |
| Diabetes mellitus |  |  |  |  |
| Negative | 371 | 66.6 | 54.2 | 0.038 |
| Positive | 134 | 55.3 | 34.4 |  |
| HBsAg |  | | | |
| Negative | 75 | 66.3 | 56.8 | 0.349 |
| Positive | 430 | 62.7 | 49.7 |  |
| Anti-HCV |  | | | |
| Negative | 497 | 62.6 | 50.3 | 0.152 |
| Positive | 8 | 66.7 | - |  |
| AFP, ng/ml |  | | | |
| <400 | 348 | 68.8 | 53.3 | 0.001 |
| ≥400 | 157 | 50.9 | 44.8 |  |
| Total bilirubin, μmol/L |  | | | |
| ≤17.1 | 353 | 62.6 | 54.3 | 0.563 |
| >17.1 | 153 | 64.9 | 42.7 |  |
| Albumin, g/L |  | | | |
| <35 | 62 | 48.3 | 28.5 | 0.004 |
| ≥35 | 443 | 65.6 | 54.2 |  |
| ALT, U/L |  | | | |
| <80 | 444 | 63.6 | 50.9 | 0.835 |
| ≥80 | 61 | 59.4 | 48.4 |  |
| GGT, U/L |  |  |  |  |
| <50 | 204 | 0.777 | 0.656 | < 0.001 |
| ≥50 | 301 | 0.541 | 0.416 |  |
| Creatinine, μmol/L |  |  |  |  |
| ≤100 | 450 | 0.626 | 0.509 | 0.681 |
| >100 | 55 | 0.683 | 0.453 |  |
| Blood urea nitrogen, mmol/L |  |  |  |  |
| ≤7 | 458 | 0.630 | 0.521 | 0.713 |
| >7 | 47 | 0.655 | 0.655 |  |
| Sodium, mmol/L |  |  |  |  |
| <140 | 157 | 0.636 | 0.559 | 0.484 |
| ≥140 | 348 | 0.630 | 0.523 |  |
| Prothrombin time, sec |  |  |  |  |
| ≤13 | 265 | 0.648 | 0.555 | 0.757 |
| >13 | 240 | 0.616 | 0.511 |  |
| Platelet count, 10^9^/L |  | | | |
| <100 | 91 | 61.7 | 44.3 | 0.639 |
| ≥100 | 414 | 63.6 | 52.5 |  |
| Ascites |  |  |  |  |
| Negative | 409 | 0.648 | 0.542 | 0.017 |
| Positive | 96 | 0.484 | 0.359 |  |
| Cirrhosis |  | | | |
| Negative | 167 | 65.2 | 58.0 | 0.800 |
| Positive | 338 | 62.6 | 48.0 |  |
| Tumor capsule |  | | | |
| Incomplete | 209 | 48.0 | 33.4 | < 0.001 |
| Complete | 296 | 74.3 | 63.3 |  |
| Macrovascular invasion |  | | | |
| Negative | 426 | 68.6 | 54.4 | < 0.001 |
| Positive | 79 | 33.0 | 16.5 |  |
| Tumor size, cm |  | | | |
| <10 | 416 | 70.2 | 55.1 | < 0.001 |
| ≥10 | 89 | 34.8 | 22.0 |  |
| Tumor number |  | | | |
| <3 | 440 | 68.0 | 55.5 | < 0.001 |
| ≥3 | 65 | 23.3 | 18.6 |  |
| Differentiation degree, *n* (%) |  |  |  |  |
| Well and moderately | 346 | 67.5 | 54.5 | 0.041 |
| poorly | 159 | 43.6 | 14.7 |  |
| Operation time, min |  |  |  |  |
| ≤180 | 363 | 65.9 | 52.5 | 0.032 |
| >180 | 143 | 56.1 | 36.2 |  |

*Calculated using data from all patients in the original cohort (without propensity score matching).

Abbreviations: AFP, alpha-fetoprotein; ALT, alanine aminotransferase; GGT, γ-glutamyl transferase; HBsAg, hepatitis B surface antigen; HCV, hepatitis C virus; OS, overall survival.
