# Supplementary material for: Impact of Diabetes Mellitus on the Prognosis of Patients with Hepatocellular Carcinoma after Curative Hepatectomy
Source: PLoS One. 2014 Dec 1;9(12):e113858. doi: 10.1371/journal.pone.0113858 (PMC4250061; doi:10.1371/journal.pone.0113858)
Supplement: Table S3 — Univariate analysis to identify factors affecting disease-free survival of patients with hepatocellular carcinoma after curative hepatectomy. (DOC) [file pone.0113858.s003.doc]

**Table S3.** Univariate analysis to identify factors affecting disease-free survival of patients with hepatocellular carcinoma after curative hepatectomy. *

| Variable | *n* | 3-yr DFS (%) | 5-yr DFS (%) | *P* |
| --- | --- | --- | --- | --- |
| Gender |  | | | |
| Male | 447 | 26.6 | 7.7 | 0.644 |
| Female | 58 | 21.3 | 0 |  |
| Age (yr) |  | | | |
| <60 | 400 | 29.8 | 8.3 | 0.588 |
| ≥60 | 105 | 5.4 | 0 |  |
| Diabetes mellitus |  | | | |
| Negative | 371 | 23.9 | 7.4 | 0.781 |
| Positive | 134 | 28.7 | 5.7 |  |
| HBsAg |  |  |  |  |
| Negative | 75 | 36.4 | 0 | 0.684 |
| Positive | 430 | 24.4 | 7.3 |  |
| Anti-HCV |  |  |  |  |
| Negative | 497 | 26.3 | 7.3 | 0.684 |
| Positive | 8 | 18.2 | 0 |  |
| AFP (ng/mL) |  |  |  |  |
| <400 | 348 | 28.7 | 10.3 | < 0.001 |
| ≥400 | 157 | 17.6 | 0 |  |
| Total bilirubin (μmol/L) |  | | | |
| ≤17.1 | 352 | 24.4 | 4.0 | 0.062 |
| >17.1 | 153 | 29.3 | 13.0 |  |
| Albumin (g/L) |  | | | |
| <35 | 62 | 25.2 | 0 | 0.321 |
| ≥35 | 443 | 26.2 | 7.5 |  |
| ALT (U/L) |  | | | |
| <80 | 444 | 26.0 | 8.8 | 0.890 |
| ≥80 | 61 | 22.2 | 0 |  |
| GGT, U/L |  | | | |
| <50 | 204 | 0.382 | 0.128 | < 0.001 |
| ≥50 | 301 | 0.190 | 0.071 |  |
| Creatinine, μmol/L |  |  |  |  |
| ≤100 | 450 | 0.244 | 0.085 | 0.233 |
| >100 | 55 | 0.318 | 0.159 |  |
| Blood urea nitrogen, mmol/L |  | | | |
| ≤7 | 458 | 0.248 | 0.078 | 0.205 |
| >7 | 47 | 0.266 | 0.177 |  |
| Sodium, mmol/L |  | | | |
| <140 | 157 | 0.146 | 0.073 | 0.739 |
| ≥140 | 348 | 0.268 | 0.074 |  |
| Prothrombin time, sec |  |  |  |  |
| ≤13 | 265 | 0.254 | 0.096 | 0.749 |
| >13 | 240 | 0.272 | 0.090 |  |
| Platelet count (10^9^/L) |  | | | |
| <100 | 91 | 23.7 | 7.9 | 0.090 |
| ≥100 | 414 | 23.9 | 7.3 |  |
| Ascites |  | | | |
| Negative | 409 | 0.217 | 0.028 | 0.816 |
| Positive | 96 | 0.325 | 0 |  |
| Cirrhosis |  | | | |
| Negative | 167 | 29.2 | 17.1 | 0.837 |
| Positive | 338 | 24.8 | 4.7 |  |
| Tumor capsule |  | | | |
| Incomplete | 209 | 14.0 | 6.0 | < 0.001 |
| Complete | 296 | 36.0 | 5.6 |  |
| Macrovascular invasion |  | | | |
| Negative | 426 | 28.6 | 8.3 | < 0.001 |
| Positive | 79 | 14.5 | 0 |  |
| Tumor size (cm) |  | | | |
| <10 | 416 | 29.5 | 8.9 | < 0.001 |
| ≥10 | 89 | 17.7 | 0 |  |
| Tumor number |  | | | |
| <3 | 440 | 28.6 | 8.5 | < 0.001 |
| ≥3 | 65 | 5.0 | 0 |  |
| Differentiation degree, *n* (%) |  |  |  |  |
| Well and moderately | 346 | 0.239 | 0.144 | 0.038 |
| poorly | 159 | 0.224 | 0 |  |
| Operation time (min) |  |  |  |  |
| ≤180 | 362 | 27.4 | 8.1 | 0.002 |
| >180 | 143 | 18.2 | 6.1 |  |

*Calculated using data from all patients in the original cohort (without propensity score matching).

Abbreviations: AFP, alpha-fetoprotein; ALT, alanine aminotransferase; DFS, disease-free survival; GGT, γ-glutamyl transferase; HBsAg, hepatitis B surface antigen; HCV, hepatitis C virus.
